# Risk Factors for SARS-CoV-2 Infection Severity in Abu Dhabi

**DOI:** 10.1007/s44197-021-00006-4

**Published:** 2021-08-23

**Authors:** Latifa Mohammad Baynouna AlKetbi, Nico Nagelkerke, Hanan Abdelbaqi, Fatima ALBlooshi, Mariam AlSaedi, Shamsa Almansoori, Ruqaya AlNuaimi, Amal AlKhoori, Aysha AlAryani, Mariam AlShamsi, Fatima Kayani, Noura Alblooshi, Shamma AlKhajeh, Jehan AlFalahi, Sumaya AlAmeri, Saeed AlDhahei

**Affiliations:** 1 Abu Dhabi Healthcare Services, 81815 Alain, United Arab Emirates; 2 UAEU, Community Medicine Department, Alain, United Arab Emirates; 3 Abu Dhabi Department of Health, Abu Dhabi, United Arab Emirates; 4 Abu Dhabi University, Public Health College, Alain, United Arab Emirates

**Keywords:** SARS-COV-2, COVID-19, Disease severity, Hospitalization, Public health, Risk factors

## Abstract

**Background:**

Prediction models are essential for informing screening, assessing prognosis, and examining options for treatment. This study aimed to assess the risk of SARS-CoV-2 infection severity in the Abu Dhabi population.

**Methods:**

This is a mixed retrospective cohort study and case–control study to explore the associated factors of receiving treatment in the community, being hospitalized, or requiring complex hospital care among patients with a diagnosis of SARS-CoV-2. Of 641 patients included, 266 were hospitalized; 135 were hospitalized and either died or required complex care, i.e., required ICU admission, intubation, or oxygen and 131 did not develop severe disease requiring complex care. The third group (“controls”) were 375 patients who were not hospitalized. Logistic regression analyses were used to study predictors of disease severity.

**Results:**

Among hospitalized patients older age and low oxygen saturation at admission were the consistent and strongest predictors of an adverse outcome. Risk factors for the death in addition to age and low oxygen saturation were elevated white blood count and low reported physical activity. Chronic kidney disease and diabetes were also associated with more severe disease in logistic regression. The mortality rate among those with less than 30 min per week of physical activity was 4.9%, while the mortality rate was 0.35% for those with physical activity > 30 min at least once a week. The interval from the onset of symptoms to admission and mortality was found to have a significant inverse relationship, with worse survival for shorter intervals.

**Conclusion:**

Oxygen saturation is an important measure that should be introduced at screening sites and used in the risk assessment of patients with SARS-CoV-2. In addition, an older age was a consistent factor in all adverse outcomes, and other factors, such as low physical activity, elevated WBC, CKD, and DM, were also identified as risk factors.

## Introduction

New pandemics such as COVID-19 caused by SARS-CoV-2 often take the world by surprise, and their impact and duration can be difficult to predict. Scientific research works hand-in-hand with public health measures to contain the spread of infections and mitigate their impact. In addition to basic epidemiology, statistical prediction models are essential for informing screening, exploring prognostic factors, establishing transmission patterns, and examining options for treatment. Models; thus, have been rapidly developed and tested to aid decision-making at points of care [1–3]. A recent meta-analysis showed that the most frequently reported predictors of the prognosis of SARS-CoV-2 infection are age, body temperature, lymphocyte count, and lung imaging features [2].

The Abu Dhabi-United Arab Emirates represent an excellent setting for this type of data management and analysis, as the healthcare providers responsible for caring for patients with SARS-COV-2/COVID-19 belong to the government hospitals and/or the Ambulatory Healthcare Service, all of which are managed by Abu Dhabi healthcare Services, SEHA. All providers use the same electronic medical record (EMR) system and have received extensive support during the pandemic to provide care for all positive cases as needed [4]. This study aimed to assess the risk of severe COVID-19 disease in the Abu Dhabi population.

## Methods

The study design was a retrospective case–control study. Cases were SARS-Cov-2 patients who required either simple hospitalization or hospitalization with more complex care while the controls were SARS-Cov-2 patients who were not hospitalized. Patients were thus stratified into three groups: (i) patients who were hospitalized and required complex care, such as required oxygenation, ICU admission, or intubation or died, (ii) patients who were hospitalized, but did not require complex care, and (iii) “control” patients who tested positive, but were not hospitalized (followed in the community).

The data sources were Electronic Medical Records reports. Cases were randomly selected patients from all Abu Dhabi hospitals admission database that included all patients admitted to any of Abu Dhabi’s SARS-CoV-2-dedicated hospitals. Control patients were randomly selected patients from the centralized data base patients attending the widely distributed screening centers established by Governmental Ambulatory Healthcare Services who tested positive for SARS-CoV-2 by PCR. The majority of these patients did not meet the UAE hospital admission criteria published by the Ministry of Health [5], and were either followed-up in the community in specially built institutions or hotels for isolation. All patients, both from hospitals (cases) and screening centers (control), were contacted by telephone for a standardized interview to collect additional data. All subjects were recruited from the two cities of Abu Dhabi Emirate, viz*.* Abu Dhabi City, and Al Ain between March 1st 2020 to the end of June 2020.

**Fig. 1:**
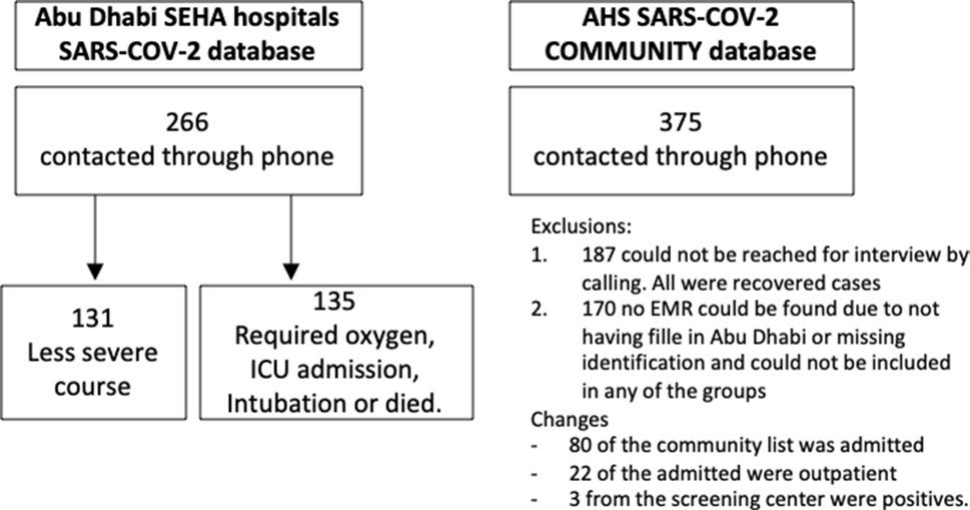
Subjects numbers and sources included in the different severity cohorts in the study

One thousand subjects from these two Abu Dhabi Emirates SARS-CoV-2 databases were randomly selected; 500 from SARS-CoV-2 screening centers database and 500 SARS-CoV-2 admitted patients. After exclusion of 187 nonrespondents for the interview call and 172 subjects with incomplete or inconsistent EMR data (Fig. 1) 641 were included in total. Eighty patients from the community list (i.e., screening centers) were also subsequently admitted to hospital, so included patients were 375 from the screening centers database who were never admitted to hospital and 266 from the admitted list (186 randomly selected from the admitted list plus 80 from the community list who were also admitted). Those who did not respond to the calls had a similar gender distribution and average age as those who responded (64% male and 36% females, 35 years mean age for both groups), but they were more likely to be non-UAE nationals (83% vs 16.5% were non-UAE nationals).

The data were collected by two methods; the data retrieved from the EMR and data collected through a standardized telephone interview conducted in Arabic and English languages as needed. The interview included demographic questions, such as age, gender, nationality, marital status, place of residence, education, and occupation; questions related to their presenting symptoms, if any, and comorbidities; and questions about health-related lifestyle habits, such as smoking, physical activities, and diet. As well, social habits of gatherings and work and travel were included. A copy of the questionnaire used for the interview is included as Supplementary Material.

The EMRs of patients were reviewed manually to ensure accuracy of data collection, consistency of the timeline of events, and correct interpretation of free text variables. The following information was collected: presenting symptoms (if any), contact history, travel history, start of first symptom, temperature, blood pressure, pulse, respiratory rate, oxygen saturation, weight, BMI, CBC (Hb, Hct, platelets, MCV, MCH), hemoglobinopathies if available, LFT (ALT, AST, AP, GGT), renal function (K, Na, eGFR), vitamin D, coagulation profile (INR, PT, PTT, D dimer), LFT, viral studies, and comorbid conditions. In addition, the outcome of infection and treatments received were collected. There were many missing values for patients who only received care in outpatient settings. For deceased patients (9), their close relatives completed the interview questions. No imputation used for the missing data.

Statistical Methods. Sample sizes was calculated on the basis of univariate comparison with 80% power and 5% significance level of a dichotomous variable, with prevalences of 30% and 50% respectively, between two groups. This yielded a minimum size of 104 patients per group. However, to allow for the presence of three groups as well as multivariate comparisons we decided to expand the sample size to the numbers we could logistically manage. The numbers we obtained were considered adequate in light of the “rule of thumb”, often criticized, that the smallest group in a logistic regression analysis should be at least 10 times the number of covariates [6].

Statistical analysis was done using SPSS version 27. Frequencies, cross tabulation in addition to logistic and ordinal regression and survival analysis. Ordinal regression was used to study severity levels reflected in either receiving treatment in the community, being hospitalized or requiring complex care in the hospital among patients with a diagnosis of SARS-CoV-2. Logistic regression (stepwise) was used to study the binary outcomes as mortality, intubation, hospitalization, and ICU admission. Kaplan–Meier curves and log rank test were used for survival analysis. All possible influencers from the variables collected were tested in the mentioned analysis. Complete case analysis was used in handling missing values. The potential impact of the missing data and their exclusion is not expected to cause systemic bias because we can expect missing data are missing completely at random (MCAR) within each severity group. With few exceptions, such as coagulation profiles in nonhospitalized patients no variable was missing more than 40%. In fact, most missingness levels were far lower.

## Results

**Fig. 2:**
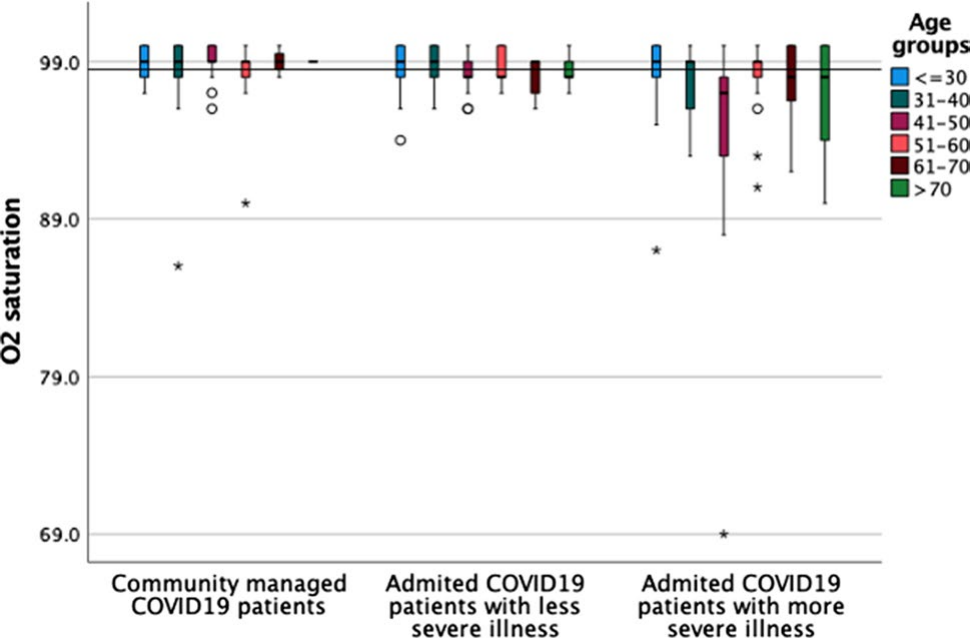
Oxygen saturation among different severity SARS-COV-2 patients

Table 1 shows the number of subjects in each group. There were 641 SARS-CoV-2 cases included in the analysis: 135 admitted and required complex care, 131 admitted with no requirement of complex care and 375 community (not hospitalized) cases. All subjects had tested positive for SARS-CoV-2-by PCR with the exception of 10 subjects with a negative test but who were diagnosed clinically, e.g., on the basis of CT findings. The mean ages among the different severity groups of patients diagnosed with SARS-CoV-2 differed markedly. The mean age was 44 years (range: 4 months–76 years) for the severe SARS-CoV-2 patients, 35 years (4 months–75 years) for those who were admitted, but not in the severe category, and 32 years (4 months–73 years) for those who were never admitted (Table 1). By logistic regression among all patients, older age, and low oxygen (Fig. 2) saturation at admission were the consistent and strongest predictors of adverse outcomes, including death, ICU admission, intubation, or requirement of oxygen (*P* = 0.004 and *P* < 0.001). Other risk factors for severe illness were being diabetic (*B* = 0.554, *P* = 0.001) and presenting with symptoms (*B* = 0.489, *P* < 0.0001) (Table 2).

**Table 1:** Characteristics of the subjects in each group

	SARS-COV-2			
	Total of all SARS-COV-2	Community	Admitted with no severe illness	Admitted with severe illness
*Age*				
< = 30	234 (36.5)	149 (39.7%)	52 (39.7%)	33 (24.4%)
31–40	184 (28.7)	138 (36.8%)	26 (19.8%)	20 (14.8%)
41–50	113 (17.6)	58 (15.5%)	28 (21.4%)	27 (20%)
51–60	55 (8.6)	24 (6.4%)	12 (9.2%)	19 (14.1%)
61–70	28 (4.4)	4 (1.1%)	8 (6.1%)	16 (11.9%)
> 70	27 (4.2)	2 (0.5%)	5 (3.8%)	20 (14.8%)
*Gender*				
Female	202 (31.5%)	114 (30.4%)	45 (34.4%)	43 (31.9%)
Male	439 (68.4%)	261 (69.6%)	86 (65.6%)	92 (68.1%)
*Marital status*				
Single	197 (30.7)	121 (33.4%)	44 (33.6%)	32 (24.1%)
Married	417 (65.1)	240 (66.3%)	84 (64.1%)	93 (69.9%)
Divorced	2 (0.3)	1 (0.3%)	1 (0.8%)	0 (0%)
Widowed	10 (1.6)	0 (0%)	2 (1.5%)	8 (6%)
*Nationality*				
Non-UAE	479 (74.7)	287 (77.8%)	105 (80.2%)	87 (64.4%)
UAE	156 (24.3)	82 (22.2%)	26 (19.8%)	48 (35.6%)
*Education*				
less than high school	192 (30)	94 (25.8%)	44 (34.9%)	54 (40%)
High School	211 (32.9)	122 (33.4%)	47 (37.3%)	42 (31.1%)
University or more	223 (34.8)	149 (40.8%)	35 (27.8%)	39 (28.9%)
*Employment*				
Employed	427 (66.6)	275 (73.3%)	80 (61.1%)	72 (53.3%)
Unemployed	37 (5.8)	20 (5.3%)	6 (4.6%)	11 (8.1%)
Housewife	75 (11.7)	25 (6.7%)	24 (18.3%)	26 (19.3%)
Retired	17 (2.7)	1 (0.3%)	4 (3.1%)	12 (8.9%)
Student	40 (6.2)	30 (8%)	4 (3.1%)	6 (4.4%)
Unskilled	24 (3.7)	8 (2.1%)	11 (8.4%)	5 (3.7%)
*Co-morbidities*				
DM	75 (11.7)	27 (7.2%)	12 (9.2%)	36 (26.7%)
Asthma	23 (3.5)	11 (2.9%)	2 (1.5%)	10 (7.4%)
Smoker	57 (8.8)	46 (12.3%)	5 (3.8%)	6 (4.4%)
No chronic illness	163 (25.4)	75 (20%)	25 (19.1%)	63 (46.7%)
CKD	19 (3)	1 (0.3%)	6 (4.6%)	12 (8.9%)
Hypertension	149 (23.2)	51 (13.6%)	41 (31.3%)	57 (42.2%)
*Symptoms*				
Asymptomatic	289 (45.1)	100 (26.7%)	95 (72.5%)	94 (69.6%)
Symptomatic	352 (54.9)	275 (73.3%)	36 (27.5%)	41 (30.4%)
History of cough	227 (35.4)	97 (25.9%)	70 (53.4%)	66 (48.9%)
History of fever	267 (41.7)	157 (41.9%)	61 (46.6%)	49 (36.3%)
CXR or CT done	357 (55.7)	98 (26.1%)	126 (96.2%)	133 (98.5%)
*Admissions*				
Hospitalization	266 (41.5)	0 (0%)	131 (100%)	135 (100%)
ICU admission	31 (4.8)	0 (0%)	0 (0%)	31 (23%)
Intubation	19 (3)	0 (0%)	0 (0%)	19 (14.1%)
Required oxygen	53 (8.3)	0 (0%)	0 (0%)	53 (39.6%)
*Physical activity *(*times per week*)				
< 1 per week	153 (23.9)	95 (25.9%)	27 (20.6%)	31 (23%)
1–2 per week	166 (25.9)	71 (19.3%)	48 (36.6%)	47 (34.8%)
3–4 per week	151 (23.6)	91 (24.8%)	34 (26%)	26 (19.3%)
5–7 per week	155 (24.2)	104 (28.3%)	21 (16%)	30 (22.2%)
> 7 per week	8 (1.2)	6 (1.6%)	1 (0.8%)	1 (0.7%)
*Contact history of SARS-COV-2*				
Never	337 (52.6)	212 (56.7%)	63 (48.5%)	62 (47%)
Once	181 (28.2)	66 (17.6%)	59 (45.4%)	56 (42.4%)
Twice	30 (4.7)	19 (5.1%)	3 (2.3%)	8 (6.1%)
Three times	11 (1.7)	8 (2.1%)	2 (1.5%)	1 (0.8%)
More than 3 times	77 (12)	69 (18.4%)	3 (2.3%)	5 (3.8%)
Travel	49 (7.6)	25 (6.7%)	14 (10.7%)	10 (7.4%)
Total	641	375	131	135

**Table 2:** Risk factors of hospitalization, complex care requirement, and adverse outcome among patients with SARS-COV-2 in Abu Dhabi

	Estimate	S.E	*P* value	Lower bound	Upper bound
*Predictors of hospitalization and complex care requirement from Ordinal regression*					
Age	0.022	0.006	< 0.001	0.011	0.33
O_2_ saturation	− 0.29	0.057	< 0.001	− 0.40	− 0.18
Presenting with symptoms	1.35	0.203	< 0.001	0.95	1.75
Diabetes mellitus	0.82	0.303	0.007	0.22	1.41

	*B*	S.E	*P* value	Exp(B)	95% C.I. for EXP(B)	
*Predictors of mortality from logistic regression*						
Age	0.257	0.107	0.016	1.293	1.05	1.593
O_2_ saturation	− 0.36	0.178	0.043	0.698	0.492	0.989
WBC	0.496	0.221	0.025	1.642	1.066	2.531
History of increased Physical activity	− 4.215	1.732	0.015	0.015	0	0.44
*Predictors of ICU admission from logistic regression*						
Age	0.068	0.016	0	1.07	1.037	1.105
O_2_ saturation	− 0.378	0.076	0	0.685	0.59	0.796
WBC	0.177	0.072	0.014	1.194	1.037	1.375
CKD	1.277	0.646	0.048	3.585	1.01	12.727
*Predictors of intubation from logistic regression*						
Age	0.064	0.018	0	1.066	1.029	1.104
O_2_ saturation	− 0.182	0.095	0.056	0.833	0.691	1.005
WBC	0.16	0.077	0.039	1.173	1.008	1.365

Out of a severe course of disease, death (*n* = 9) is clearly the worst. The risk of mortality, in *logistic regression*, among all patients increased with age (OR per year = 1.2, *P* = 0.016), low oxygen saturation (OR per 1% decrease = 0.698, *P* = 0.043), elevated white blood count (OR = 1.64 per 1 × 109/L increase, *P* = 0.025), and low reported physical activity (OR = 0.015, *P* = 0.015). The mortality rate among those with less than 30 min per week of physical activity was 5.04% as compared to a rate of 0.35% for those with physical activity > 30 min at least once per week.

Another adverse development is ICU admission. Only CKD (Chronic Kidney Disease) was found to be associated with an elevated probability of ICU admission, However, our relatively modest sample size could have caused the absence of major chronic illness as risk factor in this model.

**Fig. 3:**
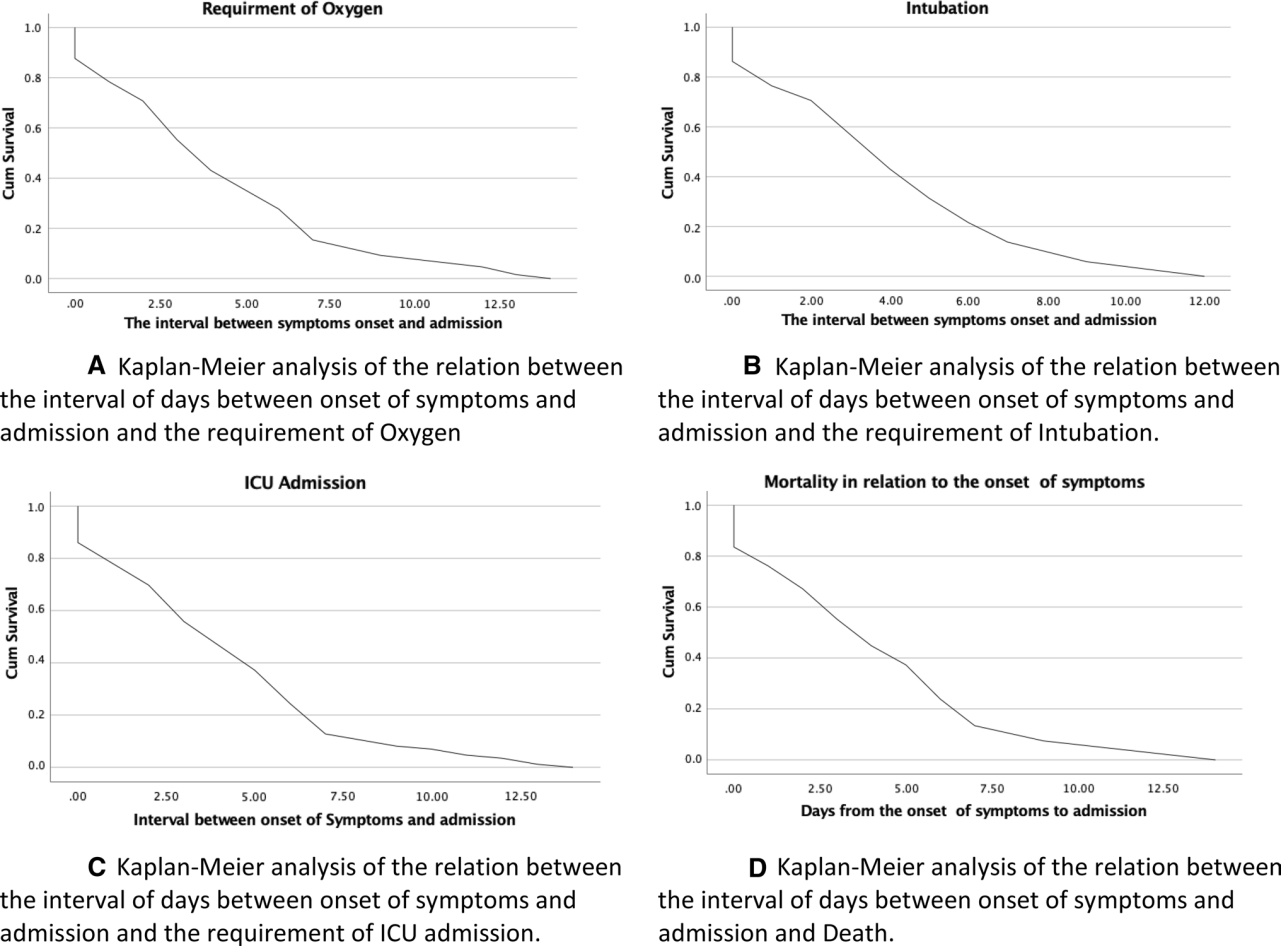
Kaplan-Meier analysis of the relation between the interval of days between the onset of symptoms among SARS-Cov-2 patients and studied outcomes

Another factor that was studied, by survival analysis, was the relationship between the interval from the onset of symptoms to admission and mortality, which yielded a significant inverse relationship, with worse survival for shorter intervals (Fig. 3).

By ordinal regression, comparing the three groups; not admitted, hospitalized, and hospitalized with complex care requirement, progress in these three levels was only determined by an older age, a lower oxygen saturation (OR (per year) = 0.02, *P* < 0.0001; OR (per 1%) = 0.29, *P* < 0.0001, respectively) and being symptomatic(OR = 1.3, *P* < 0.0001). Most of the community treated cohort (73.3%) were asymptomatic as compared to 27.5% and 30.4%) for the admitted patients’ groups. Notably, fever was a strong determinant of admission overall (OR = 4.3, *P* < 0.0001 in multivariate logistic regression.

## Discussions

The strongest risk factors for an adverse outcome or severe course of disease among hospitalized patients were advanced age, decreased oxygen saturation, and being symptomatic such as having fever, while elevated white blood count, diabetes, and chronic kidney disease were related to some of the adverse outcomes, such as death, ICU admission and intubation. These factors were similarly identified in a systematic review by Wynanuts and colleagues [2] and other studies [3, 7–9]. The present study, however, did not find a significant association between adverse outcomes and the other factors identified in other studies, such as sex, hypertension, respiratory disease, imaging findings, or BMI. However, this reflects the current literature which reports variability in prediction of outcome. Similar to this study, a recent review found no scientific support for claiming that hypertension contribute to unfavorable outcomes in COVID-19 [10]. Another study by Gebhard et al. [11] found sex as a determinant factor of survival. The variability could be attributed in this case to impact of gender-specific lifestyle, health behavior, psychological stress, and socioeconomic conditions difference between different countries. With regards to diabetes mellites Singh et al. [12] found particularly poorly controlled group to be associated with a significantly higher risk of severe COVID-19 and mortality. In this study only a diagnosis of diabetes was among the studied variables and not the glycemic control. Many other factors are being identified [13] and their expression in different setting will be of an interest.

The identification of these factors is important because they can be used, at least locally, to stratify patients and facilitate the management of patients with SARS-COV-2 in addition to adding to the gathered data internationally. Referral protocols will benefit from these findings, especially regarding stratifying the population based on the age and comorbidities and including oxygen saturation when monitoring positive cases. Especially that symptoms were more in groups with less severity. High-risk patients with SARS-CoV-2 as well seem to develop symptoms more rapidly and have a shorter interval from the development of symptoms to admission than low-risk patients, which demonstrates the variation in disease presentation and the need for well-designed prospective studies to determine the underlying etiology.

These factors may change rapidly, and different susceptible groups may emerge [14]; therefore, developing and updating such models are important for maintaining their relevance to efforts to contain the pandemic. For this model to be useful, it is necessary to conduct both regular surveillance and regular data gathering, preferably at the individual level. Risk factors identified in this study could be helpful in identifying patients at risk of severe disease much earlier than on the basis of rapidly worsening symptoms, as is often the case. A case in point is physical activity, with low physical activity a clear predictor of poor outcome.

The strength of this study is the population severity spectrum. The SARS-Cov-2 infection from any positive result in the community from SRS-Cov—2 screening as compared to all admitted patients, hospitalized, and those who required complex care. This might stimulate further studies that is well needed in the UAE as assessing how much care improvement could have been achieved by using this model vs other models, such as the 4c for example [1]. Another strength is that the study verified data from EMR through chart review to understand the diseases course and timeline of occurrence of events. Nevertheless, this limited the sample size. With the UAE having one of the lowest mortality rates in the world from SARS-Cov-2 larger sample size is needed to identify predictors of less common outcomes. Similar to other prediction models studies these results will require validation studies which are increasingly developing in different region in the world [15].

## Conclusion

Important measures were identified in this study to guide risk assessment of patients with SARS-CoV-2 at least in the UAE such as oxygen saturation, and shorter interval between onset of symptoms and hospitalization. In addition, an older age was a consistent factor in all adverse outcomes, and other factors, such as low physical activity, elevated WBC, CKD, and DM, were also identified as risk factors.

## Appendix

See Table

**Table 3:** Checklist of items to include when reporting a study developing or validating a multivariable prediction model for diagnosis or prognosis*

Section/topic	Item	Development or validation?	Checklist item	Page
*Title and abstract*				
Title	1	D; V	Identify the study as developing and/or validating a multivariable prediction model, the target population, and the outcome to be predicted	1
Abstract	2	D; V	Provide a summary of objectives, study design, setting, participants, sample size, predictors, outcome, statistical analysis, results, and conclusions	1
*Introduction*				
Background and objectives	3a	D; V	Explain the medical context (including whether diagnostic or prognostic) and rationale for developing or validating the multivariable prediction model, including references to existing models	3
	3b	D; V	Specify the objectives, including whether the study describes the development or validation of the model, or both	3
*Methods*				
Source of data	4a	D; V	Describe the study design or source of data (e.g., randomized trial, cohort, or registry data), separately for the development and validation data sets, if applicable	4
	4b	D; V	Specify the key study dates, including start of accrual; end of accrual; and, if applicable, end of follow-up	4
Participants	5a	D; V	Specify key elements of the study setting (e.g., primary care, secondary care, general population) including number and location of centres	4
	5b	D; V	Describe eligibility criteria for participants	4–5
	5c	D; V	Give details of treatments received, if relevant	4
Outcome	6a	D; V	Clearly define the outcome that is predicted by the prediction model, including how and when assessed	7
	6b	D; V	Report any actions to blind assessment of the outcome to be predicted	NA
Predictors	7a	D; V	Clearly define all predictors used in developing the multivariable prediction model, including how and when they were measured	5
	7b	D; V	Report any actions to blind assessment of predictors for the outcome and other predictors	NA
Sample size	8	D; V	Explain how the study size was arrived at	5
Missing data	9	D; V	Describe how missing data were handled (e.g., complete-case analysis, single imputation, multiple imputation) with details of any imputation method	6
Statistical analysis methods	10a	D	Describe how predictors were handled in the analyses	6
	10b	D	Specify type of model, all model-building procedures (including any predictor selection), and method for internal validation	6
	10c	V	For validation, describe how the predictions were calculated	NA
	10d	D; V	Specify all measures used to assess model performance and, if relevant, to compare multiple models	NA
	10e	V	Describe any model updating (e.g., recalibration) arising from the validation, if done	NA
Risk groups	11	D; V	Provide details on how risk groups were created, if done	NA
Development vs. validation	12	V	For validation, identify any differences from the development data in setting, eligibility criteria, outcome, and predictors	NA
*Results*				
Participants	13a	D; V	Describe the flow of participants through the study, including the number of participants with and without the outcome and, if applicable, a summary of the follow-up time. A diagram may be helpful	5–7
	13b	D; V	Describe the characteristics of the participants (basic demographics, clinical features, available predictors), including the number of participants with missing data for predictors and outcome	Table 1
	13c	V	For validation, show a comparison with the development data of the distribution of important variables (demographics, predictors and outcome)	NA
Model development	14a	D	Specify the number of participants and outcome events in each analysis	Table 2
Page7-8				
	14b	D	If done, report the unadjusted association between each candidate predictor and outcome	7–8
Model specification	15a	D	Present the full prediction model to allow predictions for individuals (i.e., all regression coefficients, and model intercept or baseline survival at a given time point)	
	15b	D	Explain how to use the prediction model	8–9
Model performance	16	D; V	Report performance measures (with CIs) for the prediction model	Table 2
Page7-8				
Model updating	17	V	If done, report the results from any model updating (i.e., model specification, model performance)	NA
*Discussion*				
Limitations	18	D; V	Discuss any limitations of the study (such as nonrepresentative sample, few events per predictor, missing data)	9
Interpretation	19a	V	For validation, discuss the results with reference to performance in the development data, and any other validation data	NA
	19b	D; V	Give an overall interpretation of the results, considering objectives, limitations, results from similar studies, and other relevant evidence	9
Implications	20	D; V	Discuss the potential clinical use of the model and implications for future research	9
*Other information*				
Supplementary information	21	D; V	Provide information about the availability of supplementary resources, such as study protocol, Web calculator, and data sets	NA
Funding	22	D; V	Give the source of funding and the role of the funders for the present study	No funding

^*^Items relevant only to the development of a prediction model are denoted by *D*, items relating solely to a validation of a prediction model are denoted by *V*, and items relating to both are denoted *D;V*. We recommend using the TRIPOD Checklist in conjunction with the TRIPOD explanation and elaboration document

From: Transparent reporting of a multivariable prediction model for individual prognosis or diagnosis (TRIPOD): the TRIPOD Statement

3.

## Declarations

### Availability of data and materials

Available on request based on the Abu Dhabi Health COVID19 Research Ethics Committee approval.
